# Influence of the postoperative inflammatory response on cognitive decline in elderly patients undergoing on-pump cardiac surgery: a controlled, prospective observational study

**DOI:** 10.1186/s12871-017-0408-1

**Published:** 2017-08-29

**Authors:** Endre Nemeth, Katalin Vig, Kristof Racz, Kinga B. Koritsanszky, Klara I. Ronkay, Fumiko P. Hamvas, Csaba Borbély, Ajandek Eory, Bela Merkely, Janos Gal

**Affiliations:** 1 Department of Anaesthesia and Intensive Therapy, Semmelweis University, P.O.B. 2, Budapest, H-1428 Hungary; 2 Heart and Vascular Centre, Semmelweis University, P.O.B. 2, Budapest, H-1428 Hungary; 3 National Institute of Neuroscience, Amerikai Street 57, Budapest, H-1145 Hungary; 4 Department of Family Medicine, Semmelweis University, P.O.B. 2, Budapest, H-1428 Hungary

**Keywords:** Cardiac surgery, Cardiopulmonary bypass, Procalcitonin, C-reactive protein, Postoperative cognitive dysfunction, Reliable Change Index

## Abstract

**Background:**

The role of non-infective inflammatory response (IR) in the aetiology of postoperative cognitive dysfunction (POCD) is still controversial. The aim of this controlled, prospective observational study was to assess the possible relationship between the grade of IR, defined by procalcitonin (PCT) changes, and development of POCD related to cardiac surgery.

**Methods:**

Forty-two patients, who were ≥ 60 years of age and scheduled for elective cardiac surgery, were separated into the low inflammatory (LIR) and high inflammatory (HIR) response groups based on their PCT levels measured on the first postoperative day. A matched normative control group of 32 subjects was recruited from primary care practice. The PCT and C-reactive protein (CRP) levels were monitored daily during the first five postoperative days. The cognitive function and mood state were preoperatively tested with a set of five neurocognitive tests and two mood inventories and at the seventh postoperative day. The Reliable Change Index modified for practice (RCIp) using data from normative controls was applied to determine the significant decline in test performance.

**Results:**

The LIR (*n* = 20) and HIR (*n* = 22) groups differed significantly in the PCT (*p* < 0.001) but not in the CRP time courses. The incidence of POCD at the first postoperative week was 35.7% in the cohort. The LIR and HIR groups did not vary in the RCIp Z scores of neurocognitive tests and frequencies of POCD (7 vs 8 cases, respectively, *p* > 0.05). Additionally, there was no difference in the mood states, anxiety levels and perioperative parameters known to influence the development of POCD.

**Conclusions:**

In this study, the magnitude of the non-infective inflammatory response generated by on-pump cardiac surgery did not influence the development of POCD in the early postoperative period in elderly patients.

## Background

Postoperative cognitive dysfunction (POCD) is known to be an important complication of cardiac and non-cardiac surgeries with marked consequences for the quality of life, work ability and intermediate-term mortality [1–4]. POCD can be characterized by a combined or specific impairment of the working memory, executive function, attention or psychomotor speed [2, 5]. The manifestation of POCD seems to be independent of age; however, it varies among age groups in both the incidence and course [5, 6]. While young and middle-aged patients experience transient cognitive decline, which recovers within a short period, the rate of POCD is up to 50% higher in elderly patients for whom the symptoms are persistent from weeks to months [5–7]. Despite the relevant amount of data published in this field in the last two decades, the explicit incidence of POCD remains debated [8]. This is due to methodological diversities in the definition of POCD or the neurocognitive tests and statistical analyses used [1, 8–10]. Hence, the interpretations of predictors and risk factors of POCD are also unclear for the same reasons. However, advanced age and the extent of surgical trauma are the most established of all investigated factors [5, 11].

The exact pathogenesis of POCD is unknown. Nevertheless, it can be supposed that POCD is a result of interactions between preoperative (patient-related), perioperative and hospital-associated factors [2, 5]. There is a strong evidence that inflammation plays a key role in the development of cognitive decline and dementia in the elderly. Elevated C-reactive protein (CRP) and interleukin-6 (IL-6) were found to be predictors of these in the general population [12–15]. The potential causative factors of POCD, surgical trauma generated inflammatory response and blood-brain barrier (BBB) disruption have been the subject of several clinical investigations in cardiac and non-cardiac surgeries in the past [5, 16, 17]. These studies confirmed a definitive inflammatory response and BBB injury after cardiac surgery in both animal and human investigations [5, 16–18]. However, the direct relationship between inflammatory response and POCD remains controversial [2, 19].

Procalcitonin (PCT) is a widely used biomarker in the diagnosis and antibiotic treatment of sepsis and its quantification has become the part of clinical practice in this field in the last 15 years [20, 21]. Nonetheless, PCT has also been described to be an appropriate indicator of non-infective postoperative inflammatory response [22–24], and it may have prognostic value for some complications related to cardiac surgery [24–26].

The aim of this controlled, prospective observational study was to assess the association between different grades of postoperative inflammatory response characterized by levels of PCT and the frequency of POCD after on-pump cardiac surgery.

## Methods

### Subjects

Patients who were aged ≥ 60 years and scheduled for elective on-pump cardiac surgery were enrolled in the cardiac surgery group. To create an age-matched, normative control group, subjects aged ≥ 60 years were recruited from primary care practice. The exclusion criteria in the cardiac surgery group, as well as in the normative controls, were significant dementia (Mini Mental State Examination score < 24), a history of cerebrovascular disease, intracranial pathology or psychiatric disease, regular treatment with benzodiazepines or anti-inflammatory drugs (e.g., steroids or non-steroids), elevated baseline PCT or CRP levels, severe left ventricle dysfunction (ejection fraction < 35%) or any kind of organ failure. Surgery or hospitalization within the last 12 months was an additional exclusion criterion in the control group.

### Anaesthetic procedure

Anaesthesia was performed using a midazolam bolus 0.05 mg/kg intravenously (IV), sufentanyl bolus 0.5 μg/kg IV, propofol bolus 1 mg/kg IV, or atracurium bolus 0.5 mg/kg IV for induction and then propofol 3–5 mg/kg/h continuous IV and sufentanyl boluses for maintenance of anaesthesia, including the cardiopulmonary bypass (CPB) period. Intraoperative monitoring of patients was based on anaesthesia standards extended with arterial blood pressure, central venous pressure, nasopharyngeal temperature and a bispectral index (BIS, Covidien LLC, Mansfield, MA USA) monitor. The depth of anaesthesia was controlled in range 45–60 of BIS for the entire surgery. CPB was provided by a roller-pump (MAQUET HL 20, MAQUET GmbH & Co. KG, Rastatt, Germany) and membrane oxygenator (MAQUET Quadrox, MAQUET GmbH & Co. KG, Rastatt, Germany). The components of CPB prime were 1200 mL of Ringer lactate, 100 mL of mannitol, and 60 mL of sodium bicarbonate 8.4%. The non-pulsatile flow rate of CPB was maintained in the range of 2.2–2.4 L/min/m^2^. The mean arterial blood pressure (MAP) was controlled with noradrenaline or glyceryl trinitrate to retain the target MAP of 60–80 mmHg during CPB. Clinical management of anaesthesia and CPB was based on institutional standards, including the temperature, metabolic targets (α-stat acid-base management) and transfusion triggers.

Continuous propofol IV infusion was administered as postoperative sedation during the mechanical ventilation period in the intensive care unit (ICU). Postoperative analgesia consisted of morphine sulphate IV boluses adjusted to the patients’ requirements and 1 g of paracetamol IV infusion (every 6 h as needed). The treatment of study patients in the ICU and cardiothoracic surgical ward did not involve benzodiazepine.

### Neuropsychological assessment

The assessment of neurocognitive functions was performed on the day before surgery and the seventh postoperative day in the cardiac surgery group by the following test battery: Mini Mental State Examination (MMSE; dementia screening); Trail Making Tests A and B (TMA and TMB, respectively; executive functions: organized visual search, planning, attention, set shifting, cognitive flexibility, and divided attention); Digit Symbol Test (DS; attention, psychomotor speed, coding task, and visual short-term memory); Stroop Colour and Word Test (cognitive flexibility and control, as well as resistance to interference). The Beck Depression Inventory (BDI; validated in native language [27]) and State-Trait Anxiety Inventory (STAI; validated in native language [28]) were used to examine mood states and anxiety levels at the same time points as the neurocognitive assessment. BDI was only performed preoperatively. Subjects in the normative control group were examined and retested after a time interval of 7 days with the same test battery and protocol. All tests were performed in a specified room separated from the cardiothoracic surgical ward and evaluated by one clinical psychologist who was blinded to the inflammatory status of the patients.

After the collection of test-retest data for each individual, the within-subject change in the performance on neurocognitive tests was measured using the Reliable Change Index modified for practice (RCIp) [29]:

1 $$ RCIp\ Z\  score=\frac{\left({\mathrm{X}}_2\hbox{--} {\mathrm{X}}_1\right)- Practice effect}{SE_{\mathrm{diff}}} $$

2 $$ {SE}_{\mathrm{diff}}=\surd \left[2{\left({SE}_m\right)}^2\right] $$

3 $$ {SE}_m={SD}_1\left[\sqrt{1-{r}_{\mathrm{xx}}}\right] $$

where *r*_*xx*_ is test-retest reliability coefficient, SD_1_ is the standard deviation of the baseline score (normative controls), SE_m_ is the standard error of measurement (normative controls), SE_diff_ is the standard error of the difference (normative controls) [29], X_2_ is the postoperative test score and X_1_ is the preoperative test score (cardiac surgery patients). The practice effect was computed by changes in the mean scores over the test-retest time interval (normative controls). A significant change was considered for an RCIp Z score ≥ ± 1.96 (*α* = 0.05) in all neurocognitive tests, including the MMSE. POCD was defined by a significant decline in ≥ two neurocognitive tests [10]. The criterion of ≥ 2 scores difference in the MMSE was used to specify the threshold of significance in the comparison of baseline cognitions in the three groups [30, 31]. MMSE scores between 28 and 30 were considered normal cognition, and the range of 24–27 was considered mild cognitive impairment [32].

### Measurement of inflammatory markers

Venous blood was collected to measure the PCT and CRP levels at the following six pre-specified time points: before the operation and then every 24 h during the first five postoperative days. Blood samples were analysed using the electrochemiluminescence immunoassay (Elecsys BRAHMS PCT, Roche Diagnostics GmbH, Mannheim, Germany) and particle enhanced turbidimetric assay (COBAS INTEGRA C-Reactive Protein Latex, Roche Diagnostics GmbH, Mannheim, Germany) techniques to quantify the PCT and CRP levels, respectively. Concentrations greater than 0.5 μg/L PCT and 5.0 mg/L CRP were considered elevated levels according to their normal values. The inflammatory response was defined as “low” for PCT ≤ 0.5 μg/L or “high” for PCT > 0.5 μg/L measured on the first postoperative day (POD).

### Statistical analysis

Continuous variables were analysed with the Shapiro-Wilk test for normality. Descriptive statistics are presented as the mean ± standard deviation for normally distributed data and the median (interquartile range) for non-normally distributed data. The unpaired *t* test and Mann-Whitney U test were used for comparisons of group means or medians. The differences in observed frequencies were determined by the χ^2^ test and Fischer’s exact test. Relationships between variables were assessed using the Spearman correlation test. To justify the sample size of this study, we calculated the statistical power of the difference between the two inflammatory responses post hoc, which was 0.7. Statistical significance was defined at *p* < 0.05 by all tests. Analysis was performed with IBM® SPSS Statistics version 23.0 (IBM® Armonk, NY, USA).

## Results

Seventy-four elderly patients with a mean age of 68 ± 6 years were recruited in the study. The details of patient characteristics and perioperative parameters can be seen in Table 1 and Table 2. The normative controls (*n* = 32) and patients in the cardiac surgery group (*n* = 42) were similar in baseline characteristics, including the age, gender and education (Table 1). Test-retest data of the normative controls used to calculate the RCIp Z scores are summarized in Table 3. We found strong test-retest reliability with reliability coefficients between 0.60 and 0.84. One of five cognitive tests (i.e., TMB) was not sensitive to practice.

**Table 1:** Baseline neurocognitive and social characteristics of the study population and normative control group

	Normative controls (*n* = 32)	Study subjects (*n* = 42)
Age (year)^a^	68 ± 7	69 ± 6
Gender (n) F / M^b^	18 / 14	20 / 22
Education (year)^c^	14 (9–16)	12 (11–12)
MMSE (score)^d^	29 (28–30)	28 (27–28)

Data are presented as the mean ± standard deviation, median (interquartile range) and number of patients

^a^unpaired *t* test

^b^χ^2^ test and Fischer’s exact test

^c^Mann-Whitney U test. *F* female, *M* male, and *MMSE* Mini Mental State Examination. There is no significant difference between normative controls and study subjects regarding age, gender and education

^d^Based on the a priori criterion of a significant difference in MMSE scores (i.e., ≥ 2 scores [30, 31]), the median MMSE scores do not differ in the normative controls and study subjects. Additionally, there is no difference in the baseline cognition between the two groups

**Table 2:** Patient characteristics and perioperative clinical data in the low and high inflammatory response groups

	LIR group *n* = 20	HIR group *n* = 22
*Preoperative parameters*		
Age (year)^a^	68 ± 6	69 ± 7
Gender (n) F / M^b^	9 / 11	11 / 11
Body mass index (kg/m^2^)^c^	29.4 (24.7–33.0)	27.2 (25.4–28.8)
Education (year)^c^	11 (11–12)	12 (11–12)
BDI (score)^c^	9 (5.3–14.3)	8.0 (5.0–10.0)
STAI-state (score)^a^	41.9 ± 10.3	41.2 ± 11.4
EuroSCORE (%)^c^	3.9 (2.8–5.7)	4.3 (2.7–7.6)
HTN (n)^b^	15	14
DM (n)^b^	5	6
CAD (n)^b^	8	9
PVD (n)^b^	3	2
COPD (n)^b^	3	4
Left ventricle EF (%)^c^	55 (55–67)	55 (50–60)
Creatinine (μmol/L)^a^	74.3 ± 12.6	84.0 ± 21.9
Antihypertensives (n)^b^	15	14
Statin use (n)^b^	11	5
Antiplatelet drug (n)^b^	11	6
Type of surgery		
CABG (n)^b^	6	5
Single valve (n)^b^	11	13
Combined (n)^b^	3	4
*Intraoperative parameters*		
Aorta cross-clamp time (minute)^a^	67 ± 22	64 ± 20
CPB time (minute)^a^	92 ± 23	91 ± 22
Rewarming time (minute)^c^	18 (14–22)	17 (10–21)
CPB temperature (°C)^c^	35.0 (34.7–35.4)	35.2 (34.7–35.5)
Operation time (minute)^a^	193 ± 27	181 ± 41
Bispectral index^a^	44.3 ± 6.0	43.1 ± 5.6
Propofol (mg/kg/h)^a^	4.1 ± 1.5	4.3 ± 1.3
Sufentanyl (μg/kg/h)^c^	0.27 (0.2–0.35)	0.28 (0.21–0.3)

The results are presented as the mean ± standard deviation, median (interquartile range) and number of patients

^a^unpaired *t* test

^b^χ^2^ test and Fischer’s exact test

^c^Mann-Whitney U test. *LIR* low inflammatory response, *HIR* high inflammatory response, *F* female, *M* male, *BDI* Beck Depression Inventory, *STAI* State-Trait Anxiety Inventory, *HTN* hypertension, *DM* diabetes mellitus, *CAD* coronary artery disease, *PVD* peripheral vascular disease, *COPD* chronic obstructive pulmonary disease, *EF* ejection fraction, *CABG* coronary artery bypass graft, and *CPB* cardiopulmonary bypass. There are no significant differences between the two groups regarding all parameters listed in this table

**Table 3:** Statistical parameters of the Reliable Change Index modified for practice measured in normative controls

Cognitive test	*r*_*xx*_	PE	SE_diff_
MMSE	0.66	0.5^***^	0.9
TMA	0.71	−7.6^**^	14.0
TMB	0.60	−9.0	29.1
DS	0.81	2.5^*^	6.1
Stroop W	0.66	1.6^***^	5.7
Stroop C	0.84	3.0^***^	4.8
Stroop CW	0.80	5.1^***^	5.2

*r*_*xx*_ test-retest reliability coefficient, *PE* practice effect, and *SE*_*diff*_ standard error of the difference. The significance of PE was tested using paired *t* test. ^*^ *p* < 0.05; ^**^ *p* < 0.01, and ^***^ *p* < 0.001; *MMSE* Mini Mental State Examination, *TMA* Trail Making Test A, *TMB* Trail Making Test B, *DS* Digit Symbol test, *Stroop W* Stroop word task, *Stroop C* Stroop colour task and *Stroop CW* Stroop colour-word task

**Fig. 1:**
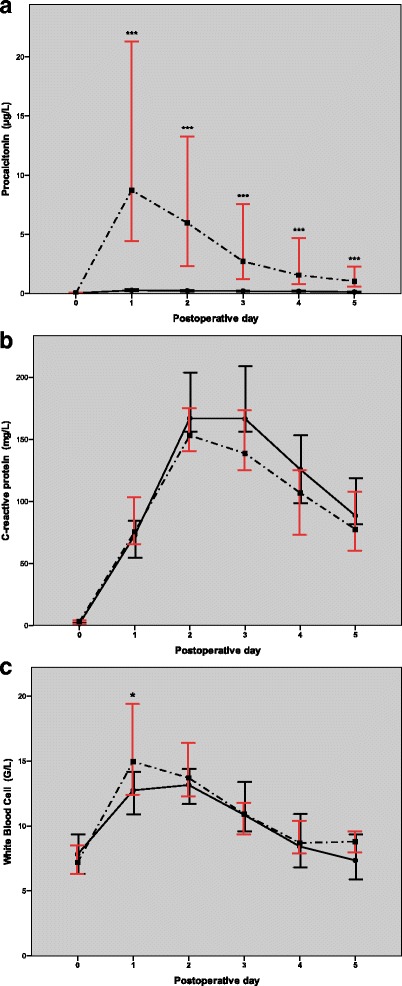
Changes in the procalcitonin (**a**), C-reactive protein (**b**) and white blood cell count (**c**) levels during the postoperative period. A *continuous line* demonstrates low inflammatory response (LIR) and a *dotted line* shows the high inflammatory response (HIR) group. *Spots* and *error bars* represent the medians and 95% confidence intervals. Significant differences between the LIR and HIR groups are demonstrated with *asterisks*: ^***^ *p* < 0.001, Mann-Whitney U test

Based on the a priori definition, cardiac surgery patients were separated into the low inflammatory response (LIR, *n* = 20) and high inflammatory response (HIR, *n* = 22) groups. While the PCT peaked at a level of 7.71 (3.90–21.52) μg/L on POD1 and then returned to 0.87 (0.48–2.50) μg/L by POD5 in the HIR group, it remained within the normal range (PCT ≤ 0.5 μg/L) with a maximum value of 0.18 (0.11–0.26) μg/L in the LIR group throughout the postoperative period (Fig. 1a). CRP reached its highest serum levels between POD2 and POD3 in both the LIR and HIR groups (166.5 (154.6–210.7) mg/L vs 138.1 (115.5–187.6) mg/L, *p* = 0.05, respectively) and did not show significant differences between the two groups during the first five postoperative days (Fig. 1b). The white blood cell (WBC) count had a significantly higher peak value on POD1 in the HIR group compared to the LIR group: 15.0 (11.9–19.5) G/L vs 12.8 (10.8–14.5) G/L, *p* = 0.012, respectively (Fig. 1c), and it showed a strong correlation with the PCT level on the first postoperative day in the HIR group (*r* = 0.67, *p* = 0.001). We did not find a correlation between the peak PCT and peak CRP values in either the HIR or LIR group (*r* = 0.10, *p* = 0.67 and *r* = − 0.42, *p* = 0.06, respectively). There were no differences between the LIR and HIR groups in the preoperative statin use, complexity of cardiac surgery, aorta cross-clamp time and CPB time (Table 2).

**Fig. 2:**
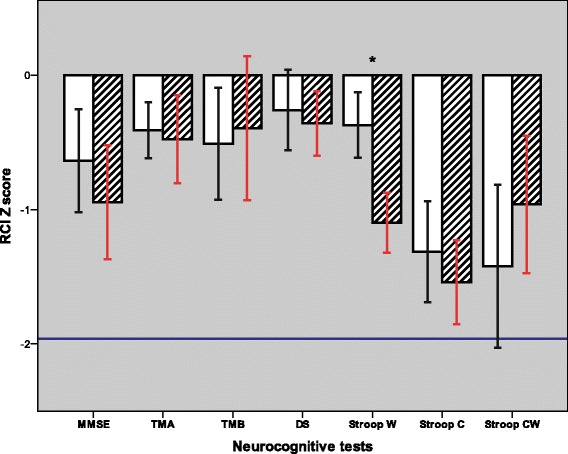
RCIp Z scores of neurocognitive tests. Z score means are demonstrated with a *blank bar* in the low inflammatory response group and *striped bar* in the high inflammatory response group. *Error bars* represent the standard error of the mean. The limit of the significant decline in performance is indicated by a continuous line at RCIp Z score of −1.96. None of the neurocognitive tests showed a significant decline at the group level. The RCIp Z scores were similar in the two inflammatory response groups, except the Stroop word task, based on the Mann-Whitney U test. Significant differences between the LIR and HIR groups are demonstrated with *asterisks*: ^*^ *p* < 0.05. MMSE = Mini Mental State Examination; TMA = Trail Making Test A; TMB = Trail Making Test B; DS = Digit Symbol test; Stroop W = Stroop word task; Stroop C = Stroop colour task; and Stroop CW = Stroop colour-word task

Seventy-four percent of cardiac surgical patients (*n* = 31) had a significantly decreased performance in at least one cognitive test according to their RCIp Z scores 1 week after surgery. Cognitive flexibility and control were the most vulnerable tasks to cardiac surgery, as demonstrated by the Stroop word-colour task results. Additionally, 38.1% of patients (*n* = 16) had a significant decline in this test. Based on the a priori definition of the POCD (i.e., a significant decline at least in two neurocognitive tests), 15 of 42 patients (35.7%) met the criteria of POCD. The RCIp Z scores of each neurocognitive test were similar in the two inflammatory response groups (Fig. 2). Hence, the frequencies of POCD in the LIR group did not vary from that in the HIR group observed at the first postoperative week (7 cases vs 8 cases, respectively). We did not find differences between the LIR and HIR groups in the preoperative BDI scores; pre- and postoperative levels of anxiety; and intraoperative parameters, including the CPB time, body temperature on-CPB, rewarming time on-CPB, operation time, BIS values, propofol and sufentanyl requirements (Table 2). We registered similar postoperative ventilation times in the two inflammatory response groups, LIR: 5.3 (4.4–8.3) hour vs HIR: 5.7 (3.3–7.9) hour, *p* = 0.60 and the morphine requirement during the first 24 h were also comparable, LIR: 0.11 (0–0.2) mg/kg vs HIR: 0.11 (0–0.3) mg/kg, *p* = 0.38. Infection or sepsis and delirium did not develop among cardiac surgical patients in the postoperative period. Details of the postoperative outcome are summarized in Table 4.

**Table 4:** Postoperative outcome parameters in the low and high inflammatory response groups

	LIR group (*n* = 20)	HIR group (*n* = 22)
Ventilation time (hour)^a^	5.3 (4.4–8.3)	5.7 (3.3–7.9)
Transfused patients (n)^b^	6	6
PRC (unit)^a^	0 (0)	0 (0–1)
Respiratory failure (n)^b^	3	2
New onset AF(n)^b^	5	4
Acute kidney injury (n)^b^	2	5
STAI-state (score)^c^	36.7 ± 8.8	38.2 ± 8.1
POCD (n)^b^	7	8
Length-of-ICU-stay (hour)^a^	24 (24–46)	23 (22–60)
Length-of-hospital-stay (day)^a^	10 (8–17)	11 (9–13)
In-hospital death (n)	0	0

Data are presented as the median (interquartile range), number of patients and mean ± standard deviation

^a^Mann-Whitney U test

^b^χ^2^ test and Fischer’s exact test

^c^unpaired *t* test; *LIR* low inflammatory response, *HIR* high inflammatory response, *PRC* packed red cells, *AF* atrial fibrillation, *STAI* State-Trait Anxiety Inventory, *POCD* postoperative cognitive dysfunction, and *ICU* intensive care unit. There are no significant differences between the two groups regarding all parameters listed in this table

## Discussion

This study was designed to explore whether the grade of postoperative non-infective inflammatory response generated by CPB surgery directly affects the development of POCD. We distinguished between two different inflammatory response levels (i.e., LIR and HIR) of our elderly patients and applied a strict definition of POCD based on statistical change criteria. With the exclusion of confounding factors, our study did not find a direct relationship between the magnitude of non-infective inflammatory response and incidence of POCD.

### Defining the inflammatory response related to cardiac surgery

Cardiac surgery is frequently accompanied by non-infective systemic inflammatory response syndrome (SIRS) induced by surgical trauma, CPB, organ ischaemia-reperfusion injuries, a change in the body temperature and endotoxin release [33, 34]. This genetically influenced, complex inflammatory process – depending on its magnitude – can result in multi-organ dysfunction, increasing the risk of mortality in the postoperative period [33, 35, 36]. Numerous inflammatory mediators have been investigated to characterize the inflammatory responses related to cardiac surgery with the purpose of using them as prognostic markers of the complications and outcome [37]. PCT is one of the accepted and widely used inflammatory markers, and it is also applied for follow-up in the cardiac surgery setting [24, 25]. Its use is based on the assumption that proinflammatory cytokines can contribute to PCT induction after surgery [24, 26]. In the case of non-infective SIRS, the PCT concentration increases in the first 24–48 h after cardiac surgery up to its maximum level and returns to the baseline range in the subsequent days [24, 26]. The peak level of PCT seems to be associated with the extent of surgical procedure, duration of aortic cross-clamp and CPB, and the length of operation [24]. Constantly elevated PCT levels in the postoperative period suggest ongoing inflammation due to a possible septic process [20, 24, 38].

In our study, we followed up PCT as a primary and CRP as a secondary inflammatory marker to distinguish the grade of the inflammatory response in the perioperative period. We observed significant differences in the magnitudes of PCT changes in the individuals who were clearly isolated to the LIR and HIR groups. While the main characteristics of the PCT time course in the HIR group were consistent with previous reports as it reached a peak value at 7.71 μg/L on POD1 [35, 38–40], PCT did not exceed the cut-off value of 0.5 μg/L at any time-point in the LIR group (Fig. 1a). Despite the markedly divergent PCT kinetics in the LIR and HIR groups, they did not differ in the perioperative parameters that are supposed to influence the inflammatory response and course, such as the preoperative statin use, duration of aortic cross-clamp and CPB, length of operation, propofol requirements, number of transfusions and postoperative infection [33, 41–44] (Table 2). We also investigated the possible factors of immune priming. Coexisting chronic diseases, such as diabetes mellitus (DM) or peripheral vascular disease (PVD), are frequently accompanied by a pro-inflammatory state, which possibly amplifies the inflammatory response in the postoperative period [2]. However, our comparative analysis did not show differences in DM and PVD between the LIR and HIR groups. These results are similar to the findings of previous studies [36, 45], supporting that an evolving complex inflammatory response to stimuli of CPB surgery is primarily determined by the individual reactivity of cytokines and the proinflammatory-anti-inflammatory balance. In the present study, we found a completely different behaviour of the CRP level compared to PCT. The results published elsewhere show that CRP increases after cardiac surgery irrespective of the extent of the surgery or presence of SIRS [24, 26, 39]. This fact makes the interpretation of the CRP time course uncertain. Our results agree with these findings because the peak CRP did not correlate with peak PCT, and it was markedly elevated in all subjects of the cohort and did not vary between the LIR and HIR groups during the postoperative period. Interestingly, the WBC count did correlate with PCT rather than with CRP in the HIR group. Hence, our data confirm that PCT follow-up is appropriate to discriminate the grade of the non-infective inflammatory response related to cardiac surgery.

### Role of the inflammatory response in the development of POCD after cardiac surgery

The occurrence of POCD was 35.7% in this study based on RCIp analysis [29] of neurocognitive tests and the definition of decline in at least two tests. Instead of frequently used fixed cut-off methods (i.e., 20% or 1–2 SD change) [8, 29], we applied RCIp involving the age-matched healthy non-surgical control group to determine the incidence of POCD. RCIp employs statistical change criteria –corrected for measurement error and mean practice effect– to estimate the valid change of performance on a neurocognitive test [8, 29, 46]. There are only a few comparable publications in the literature that apply statistical change criteria methods in a cardiac surgery setting [29, 47–49]. The observed POCD incidence in our elderly group of patients is in line with their findings (36 vs 33–43%, respectively), which supports the conclusion of a recent investigation of Raymond et al. [29] Analyses that use statistical change criteria can considerably contribute to valid estimation of POCD as they minimize the risk of both overestimation and underestimation of decline in the test performance [8, 29].

The main result of this study was that a direct relationship has not been revealed between the degree of PCT elevation and decline in any neurocognitive test or early POCD. The secondary analysis of our data validated this result as perioperative mood states, and predisposing factors of POCD [2, 6, 47, 50] were similar in the two inflammatory response groups defined by the PCT levels (Tables 2 and 4). Furthermore, we did not observe postoperative complications that affect cognitive function. Numerous investigations have focused on the link between proinflammatory cytokines and cognitive dysfunction related to cardiac surgery in the last two decades [2, 17]. Using animal models, Cibelli et al. [51] and Terrando et al. [52] described a potential link between systemic and hippocampal inflammation through the TNF-α, IL-1β and NF-κB pathways, and the impairment of memory as a consequence of the former processes. Jungwirth et al. also confirmed significant cerebral expression of NF-κB in the hippocampus after CPB surgery in a high-quality randomized controlled animal study; however, it was not associated with the neurocognitive outcome [53]. Clinical trials applying arbitrary cut-off criteria for POCD definition in either the cardiac or non-cardiac surgery setting concluded conflicting results on the relationship between pro-inflammatory cytokines and POCD [54–56]. In a recent investigation of elective coronary artery bypass patients, Hudetz et al. demonstrated that short- and medium-term cognitive dysfunction was related to elevated postoperative IL-6 and CRP levels [57]. Their results were based on the POCD definition involving data from the normative population and Z score [57]. By contrast, most recently published large randomized clinical trials have conflicting results on the incidence of POCD or postoperative delirium when they used pharmacological anti-inflammatory treatment (i.e., dexamethasone or methylprednisolone) during non-cardiac and cardiac surgery [19, 58, 59]. This result might strengthen earlier assumptions that factors other than the grade of the inflammatory response play key role in the pathogenesis of POCD [53, 56]. Our results support this concept because we could clearly demonstrate that the incidence of POCD measured in this study does not depend on the magnitude of the inflammatory response.

### Limitations

Our investigation has several limitations. First, the study was conducted in a single centre, which influenced the sample size over the study period. The description of the postoperative inflammatory response was based on the PCT, CRP and WBC count measurements, and we did not involve pro-inflammatory cytokines in the analysis for further specifications. Our study did not strictly adhere to the Statement of Consensus on Assessment of Neurobehavioral Outcomes after Cardiac Surgery [60] in terms of the applied neurocognitive tests. This study was designed for the short interval outcome measure that aimed to assess the early changes in cognitive function after cardiac surgery, which restricts the interpretation of our results. Considering the post hoc statistical power of this observational study, our presented results are preliminary results, while these data are not confirmed by further investigations.

## Conclusions

Despite the significant difference in the magnitude of the non-infective inflammatory response induced by CPB surgery, the incidence of POCD was similar in the LIR and HIR groups of our elderly patients when POCD was measured in a strict methodological framework. The role of the inflammatory response in the pathogenesis of POCD needs to be cleared by further investigations.

## Abbreviations

BBB: Blood-brain barrier
BDI: Beck Depression Inventory
CPB: Cardiopulmonary bypass
CRP: C-reactive protein
DM: Diabetes mellitus
DS: Digit Symbol Test
HIR: High inflammatory response
ICU: Intensive Care Unit
IL-6: Interleukin-6
IV: Intravenously
LIR: Low inflammatory response
MAP: Mean arterial pressure
MMSE: Mini Mental State Examination
PCT: Procalcitonin
POCD: Postoperative cognitive dysfunction
POD: Postoperative day
PVD: Peripheral vascular disease
RCIp: Reliable Change Index modified for practice
*r*_*xx*_: Test-retest reliability coefficient
SD: Standard deviation
SE_diff_: Standard error of the difference
SE_m_: Standard error of measurement
SIRS: Systemic inflammatory response syndrome
STAI: State-trait anxiety inventory
TMA: Trail-Making A
TMB: Trail-Making B
WBC: White blood cell
